# Prediction of the performance of pre‐packed purification columns through machine learning

**DOI:** 10.1002/jssc.202100864

**Published:** 2022-03-20

**Authors:** Qihao Jiang, Sohan Seth, Theresa Scharl, Tim Schroeder, Alois Jungbauer, Simone Dimartino

**Affiliations:** ^1^ Institute of Bioengineering School of Engineering The University of Edinburgh Edinburgh UK; ^2^ School of Informatics The University of Edinburgh Edinburgh UK; ^3^ Austrian Centre of Industrial Biotechnology Vienna Austria; ^4^ Institute of Statistics University of Natural Resources and Life Sciences Vienna Vienna Austria; ^5^ Repligen GmbH Ravensburg Germany; ^6^ Department of Biotechnology University of Natural Resources and Life Sciences Vienna Austria

**Keywords:** asymmetry, machine learning, plate height, porous media, pre‐packed columns

## Abstract

Pre‐packed columns have been increasingly used in process development and biomanufacturing thanks to their ease of use and consistency. Traditionally, packing quality is predicted through rate models, which require extensive calibration efforts through independent experiments to determine relevant mass transfer and kinetic rate constants. Here we propose machine learning as a complementary predictive tool for column performance. A machine learning algorithm, extreme gradient boosting, was applied to a large data set of packing quality (plate height and asymmetry) for pre‐packed columns as a function of quantitative parameters (column length, column diameter, and particle size) and qualitative attributes (backbone and functional mode). The machine learning model offered excellent predictive capabilities for the plate height and the asymmetry (90 and 93%, respectively), with packing quality strongly influenced by backbone (∼70% relative importance) and functional mode (∼15% relative importance), well above all other quantitative column parameters. The results highlight the ability of machine learning to provide reliable predictions of column performance from simple, generic parameters, including strategic qualitative parameters such as backbone and functionality, usually excluded from quantitative considerations. Our results will guide further efforts in column optimization, for example, by focusing on improvements of backbone and functional mode to obtain optimized packings.

## 1 INTRODUCTION

Pre‐packed chromatography columns are widely employed in process development and biomanufacturing. Their biggest advantage is to take away the burden of costly and time‐consuming packing procedures and associated validation protocols, ultimately ensuring a consistent product [1, 2, 3, 4]. The production of pre‐packed columns should be simple, cost‐effective, and robust over the long term (decades) to ensure consistent quality of columns.

The performance of pre‐packed columns is assured by the manufacturer before the sale, with packing quality measured in terms of the height equivalent to a theoretical plate (HETP) and asymmetry. Both parameters are calculated from the response of the column following a pulse injection of a non‐binding tracer, that is residence time distribution (RTD) experiments. The HETP corresponds to the column length over the number of theoretical plates (*N*), with efficient columns characterized by relatively large *N* and small HETP values. According to the general rate model, the RTD response of a “well‐packed” column is a symmetrical Gaussian peak. To better assess packing quality, RTD experiments are usually run under conditions for which hydrodynamic dispersion is the dominant contribution to mass transfer (negligible intraparticle mass transfer, no adsorption). Under these conditions (reduced velocity of about 1–10), the HETP The minimum HETP value theoretically depends only on the properties of the tracer, the velocity of the mobile phase, and the size of the chromatographic particles [5]. However, the general rate model is unable to capture how the HETP is influenced by key factors of practical relevance such as column size (column diameter and length) or ease of packing across different chromatographic resins [6]. For example, Scharl et al. [7] qualitatively discussed the importance of material backbone on the packing quality of a range of pre‐packed columns. Deviations from symmetrical peaks are often observed in practice, with peak fronting or tailing associated with a number of non‐idealities such as wall effects, inhomogeneous packing, inhomogeneous distribution of the solute over the bed at the column inlet/distributor and/or at the outlet/collector, and dispersion in the extra column volumes [8, 9, 10, 11, 12]. Such deviations are measured through the asymmetry, an empirical parameter used to quantify the degree of peak skewness and employed to assess packing quality in tandem to the HETP [13].

Mathematical models to predict column performance and chromatographic processes, including the general rate model, are generally based on first principles. In particular, they include details of mass transfer phenomena and binding kinetics to describe peak profiles and breakthrough curves [14, 15]. While the predictive power of these models is often excellent, they require extensive calibration efforts through independent experiments, for example, to determine key model parameters such as mass transfer and kinetic coefficients [16, 17]. Flow non‐idealities such as wall effects and distribution/collection of the fluid at the column inlet/outlet also require independent experiments for them to be accounted for in the models. These additional experiments are specific to the chromatographic system (external column volumes) and column (diameter, length) employed, therefore cannot be extrapolated to different systems or different columns. Finally, such models based on first principles do not take into account qualitative variables such as resin backbone and functional chemistry by design.

Machine learning (ML) could represent an alternative modeling approach to analyze and predict column performance. The main advantage of ML is the ability to extract information from large data sets using no or only minimum assumptions, eventually determining generalizable predictive patterns between multiple inputs (including quantitative, qualitative, and categorical parameters), and the output variables [18, 19]. A number of algorithms, for example, support vector machines, decision trees, gradient boosting, and deep neural networks have been developed over the years, and have proved their ability in dealing with complex data problems in a practical manner [20, 21]. ML has been applied to chromatography systems, with many successful applications, for example, in peak observation [22, 23, 24], retention modeling [25, 26, 27, 28], process optimization[29, 30, 31], and real‐time process monitoring [32, 33]. The main challenge associated with the application of ML is the availability of very large experimental data sets for the ML algorithm to draw meaningful correlations.

In this work, we consider a large data set of around 25 000 quality assurance experiments of pre‐packed columns manufactured and tested under standardized conditions for a period of over 10 years [7]. We first examine the time series of the data set using correlation and autocorrelation analysis to ensure the data are self‐consistent and time‐independent. We then employ ML methods to find a correlation between column performance (measured in terms of HETP and asymmetry) and qualitative column variables, namely resin backbone, functionalization chemistry, column size (length and diameter), and particle size. The results are finally commented on in relation to the main key variables affecting column performance.

## 2 MATERIALS AND METHODS

### 2.1 Experimental data set

The data set employed in this work is a subset of that previously employed by Scharl et al consisting of 24 951 quality control runs of pre‐packed small‐scale columns over a period of about 10 years [7]. The data contain relevant column parameters (i.e., column length and diameter, particle size, backbone material, functional mode, and date of testing) together with reduced HETP (*h*) and asymmetry ($A_{s}$). Column diameter and length ranged between 5 and 11.3 mm and 10 and 100 mm, respectively, while particle diameter varied between 15 and 400 μm. 2232 experimental runs (approximately 10%) were removed from the original data set as they lacked one or more column parameter inputs, reducing the data set to a total of 22 359 tests. Columns with the same attributes were manufactured and tested more than once over the 10 years monitored, with some popular types examined hundreds of times (for example, see Table S1). All experiments having the same set of input features were treated as a single entry, with h and $A_{s}$ averaged over the available runs for that column type. This step was necessary to prevent data leakage in the ML model, that is, the use of the same column type in both the training and testing data sets (see Section 2.3), as well as to prevent overfitting of the most popular column types over the ones infrequently produced. The standard error for h and $A_{s}$ was always lower than 10%, indicating that the average h and $A_{s}$ are representative output indicators of column performance for any given column type. After the averaging process, the data set contained a total of 546 independent runs (num=546).

All columns used to generate the data set were packed by slurry packing under vibration following a standardized procedure developed by the packing company (Atoll, now Repligen). The packing quality of the columns was evaluated using a standardized experimental setup and experimental protocol as reported in Scharl et al [7]. Briefly, the response of the column following an acetone or sodium nitrate injection was measured, and the resulting chromatographic peak was analyzed to extract h and $A_{s}$. This simple experiment allowed to isolate the contribution to band broadening associated with hydrodynamic dispersion (which in turn depends on packing quality and extra column dispersion) as the tracers employed are both non‐retained (i.e., zero retention factor), with practically the same diffusion coefficients ($1.2\times 10^{-5}$ and $1.3\times 10^{-5}$ cm^2^/s for acetone [34] and sodium nitrate [35], respectively), and tested under reduced velocities comprised between 1 and 20 for which the minimum HETP is obtained [14].

### 2.2 Extreme gradient boosting

Extreme gradient boosting (XGBoost) is a scalable ML system for tree boosting [36]. XGBoost is a decision‐tree‐based ensemble learning method [37] that provides a systematic solution to a given problem by combining the predictive power of several different or same ML algorithms. The algorithm used in XGBoost is the Classification and Regression Tree [38] which employs a binary tree that can be constantly segmented by data features, thus enabling dynamic growth of the tree. The characteristics of the input data will eventually fall into the leaf nodes n the tree, where each leaf node corresponds to a specific score, and the sum of the scores in all the leaf nodes computes the final prediction value of a certain feature, for example, *h* or *A_s_*.

Essential details of the mathematical formulation of the XGBoost model are presented in the following, with additional details in the SI. For a given data set with *n* examples and *m* features $D=\left\{\left(x_{i},y_{i}\right)\right\}\;|(\;|D|=\;n,x_{i}\in R^{m},y_{i}\in R)$, the tree ensemble model uses K additive functions to predict the output.

(1) $$\;{\hat{y}}_{i}=\sum_{k=1}^{K}f_{k}\left(x_{i}\right),f_{k}\in F\;$$

where $F\;=\left\{f\left(x\right)=w_{q(x)}\right\}\;\left(q:R^{m}\to T,w\in R^{T}\right)$ is the space of the regression trees. The q represents the structure of each tree that maps an example to the corresponding leaf index. T is the number of leaves in the tree. Each $f_{k}$ corresponds to an independent tree structure q and tree weights w. More mathematical details can be found from the original XGBoost paper [36].

The regularized objective function defined for XGBoost, L, can be written as:

(2) $$L\;=\sum_{i}l\left({\hat{y}}_{i},y_{i}\right)\;+\sum_{k}\Omega \left(f_{k}\right)$$

(3) $$\Omega \;\left(f\right)=\;\gamma \cdot T+\frac{1}{2}\lambda {\left\|w\right\|}^{2}$$

Here l is a differentiable convex loss function that measures the difference between the prediction ${\hat{y}}_{i}$ and the target $y_{i}$. The second Ω term prevents unnecessary large trees by penalizing the complexity of the model, in turn avoiding overfitting. The additional regularization term $\frac{1}{2}\lambda {∥w∥}^{2}$ helps smooth the final learned weights. The shrinkage parameter γ is an additional design to prevent over‐fitting. The γ is utilized to multiply the score of each leaf node by a reduction weight during the iteration, which ensures that the influence of each tree is not too large, leaving more space for the tress generated later to optimize.

XGBoost is also used to determine the relative importance of the input features. The definition of relative importance is followed by the study of H. Friedman [39]. For a tree model whose number of terminal nodes is J, the relative importance of a given input feature, I, is calculated by the sum of the corresponding empirical improvements, $i^{2}$, with t referring to a non‐terminal node and $v_{t}$ acting as splitting variable for that node. The $i^{2}$ term is determined from the two sub‐region $R_{l}$ and $R_{r}$, where ${\bar{y}}_{l}$ and ${\bar{y}}_{r}$ are the response means, respectively, and $w_{l}$ and $w_{r}$ are the corresponding sums of the weights. In Python, the contribution of each input feature can be automatically transferred into the percentage version.

(4) $$I_{j}^{2}\;\left(T\right)=\sum_{t=1}^{J-1}i_{t}^{2}\left(v_{t}=j\right)$$

(5) $$i^{2}\;\left(R_{l},R_{r}\right)=\frac{w_{l}w_{r}}{w_{l}+w_{r}}\;{\left({\bar{y}}_{l}-{\bar{y}}_{r}\right)}^{2}$$

### 2.3 Data pre‐processing and model implementation

Functional modes and backbone are two categorical features that cannot be operated by many ML algorithms directly. One‐hot encoding was applied to transfer them into numerical values [40], with each feature normalized between 0 and 1. All other numerical parameters were also normalized between 0 and 1 before input into the ML model as most ML algorithms perform better or converge faster with features on a relatively similar scale [41].

An XGBoost regression model was created in Python 3.6 combining i) GridSearchCV (ten‐folds) to select and determine the model's hyper‐parameters (for example, learning rate, maximum tree depth, and minimum child weight) [42] and ii) XGBRegressor as the main package to process our data set [43]. The whole data set was then separated randomly into a training set (66.7%) and testing set (33.3%), with the training set utilized for training the ML model and the testing set used for inspecting the final model accuracy. Mean absolute error (MAE) [44] was used as the evaluation metric during model training. The final prediction precision of the model is reported by the mean absolute percentage error (MAPE) between the prediction results and the testing data set. The overall model prediction capability remained the same when changing initial seeding to randomly generate different training and testing data sets.

## 3 RESULTS AND DISCUSSION

The main goal of this study was the identification of a general relationship between column parameters (column length, column diameter, particle diameter, functional mode, backbone material) and chromatographic performance (reduced HETP, hand peak asymmetry, $A_{s}$) using ML algorithms as an alternative to classical rate models for chromatography. Classical rate models are derived from first principles and thus tend to be the preferred choice when it comes to the modeling of chromatographic separations. However, some of the parameters entering rate models often are either determined through empirical expressions (for example, the Wilson Geankopolis correlation for the estimation of the mass transfer coefficient [45]) or simply adjusted to best‐fit experimental results (for example, diffusion or dispersion coefficients [46]).

The introduction of a certain degree of empiricism in physical models is necessary to capture important elements of the model hard to describe in mathematical terms. For example, the 3D configuration of chromatographic beds deviates from the theoretical close random packing limit [47], with the resulting bed arrangement strongly influenced by attributes linked to the material and column properties (for example, Young modulus, friction factor, and wall roughness) as well as the packing procedure itself [47, 48]. For example, Knox demonstrated that hydrodynamic dispersion in columns packed with smooth non‐porous glass beads is smaller than those measured in columns packed with porous glass [49]. Knox explained this result in terms of bed homogeneity and speculated that smooth glass particles are able to form relatively regular packings, while porous glass particles are affected by greater interparticle friction forces, in turn resulting in particle bridging and the formation of pockets where local mixing occurs. These insights were demonstrated experimentally by Patel et al. [50], who confirmed that the A term in the van Deemter equation is primarily associated with radial heterogeneities in the bed. On the opposite front, Malkin et al. showed that submicrometer silica particles tend to pack close to the limit of a face‐centered cubic arrangement [51], resulting in reduced plate heights below 1. Khirevich et al. also reported that the local microscopic disorder in packings was highly correlated with eddy dispersion, directly affecting column performance [52]. Along the same line, Gritti et al [53] reported the outstanding performance of columns packed with core‐shell particles, partly attributing these results to the propensity that these particles have to create homogeneous beds. More recent studies on 3D printed ordered beds further confirm the advantages of perfectly ordered packing, with simulated reduced plate heights below 0.1 for specific arrangements (for example, octahedral particles in simple cubic configuration) of non‐porous stationary phases under non‐retained conditions [54].

The concept of “goodness of packing” as proposed by Knox is strongly correlated to the A term of the van Deemter equation [55], with lower A values associated to lower reduced plate heights and hence higher chromatographic efficiency. According to the general rate model for chromatography, the A term can be expressed as [16]:

(6) $$A\;=\;2\chi d_{p}$$

or in dimensionless terms:

(7) a=2χ

where $d_{p}$ is the average particle diameter and χ is the dispersivity of the stationary phase. The dispersivity is a characteristic determined by the hydrodynamics in the column, in turn, defined by the type of particles and their packing. For a given column, the dispersivity can be determined through estimation of the plate height under conditions suppressing both axial diffusion (i.e., large velocity, negligible B term) and mass transfer and kinetic resistances (i.e., injection of a small, fast diffusing non‐adsorbing tracer, negligible C‐term) for which the van Deemter equation reduces to:

(8) h=a=2χ

While this equation represents a relatively rapid method to assess the hydrodynamic properties of a given column, the lack of correlations for the estimation of the dispersivity coefficient represents a limitation to predict band broadening due to axial dispersion. In particular, there exist no quantitative method to assess how the dispersivity depends on different column properties such as:

‐ backbone material and functional mode, closely related to the propensity of theparticles to generate regular packing;
‐ column and particle diameters, that is, the column to particle ratio, in turn determining the importance of non‐homogeneities close to the column wall with respect to the rest of the column volume;
‐ column length and column diameter, which are associated with both bed compressibility [56], as well as defining the relative influence of extra‐column dispersion effects, for example, due to non‐uniformity of the velocity profile resulting from non‐idealities in the extra‐column volumes.

Fronting or tailing deviations from the ideal symmetrical peak are often observed in chromatographic practice, negatively impacting the separation performance. Such deviation is often quantified through the asymmetry factor, $A_{s}$, defined as the ratio between the width of the tailing end and the peak front at 10% peak height [57, 58]. Large asymmetry factors are associated with the heterogeneity of the column packing [59, 60], making $A_{s}$ another excellent descriptor for “goodness of packing”. However, search for a quantitative relationship between asymmetry and column parameters has been elusive so far. In this context, ML is an excellent tool to extract poorly understood links between variables such as the column input parameters and the asymmetry factor.

The data set of pre‐packed column performance offers an opportunity to quantitatively analyze the dependence of the dispersivity on a range of qualitative and quantitative column attributes. The two performance parameters, *h* and *A_s_*, are measured from the experimental response of an injection of a small non‐retained tracer (acetone or sodium nitrate). Same experimental and data analysis methods were used to generate the entire data set [7]. Only resins intended to separate proteins or other larger biomolecules were tested, ensuring much larger pores than that of the tracers. Such conditions ensure only the hydrodynamic dispersion is captured in the experiments and that the Van Deemter equation can be simplified into Equation (8).

In short, we propose here to employ ML as a powerful alternative to traditional chromatographic models to investigate a correlation between the different column input parameters and the output performance parameters. ML is especially valuable in this context given the complexity of the problem described and the qualitative nature of some of the relevant variables such as column backbone and functional mode. ML is also able to suggest the relative importance of the different inputs with respect to the outputs, thus helping the identification of the key descriptors for the performance parameters.

### 3.1 Time series of reduced plate height and asymmetry

Column performance can change over time due to variations in the manufacturing line, for example, improvement in the packing procedures, change of suppliers of raw materials, and aging of the production line. Scharl et al. qualitatively observed that the plate height of the prepacked columns tested was stable over 10 years [7]. However, any interdependence between *h* and *A_s_* with time needs to be either identified or excluded in quantitative terms to avoid any input bias to the ML model. In other words, it is first necessary to determine if time represents an input variable to the ML model, as well as if sampling and testing of the columns changed significantly over time. Autocorrelation and partial autocorrelation analysis was employed onto the data set to address these two aims, respectively. In particular, the autocorrelation function (acf) aims to detect cross‐similarities of a signal with itself at a different time (time lag) [61]. In this context, acf helps detect changes in the manufacturing line and in the quality assurance protocols employed over time. The partial autocorrelation function (pacf) instead aims to identify the possibility of confounding variables that are correlated to both variables [62]. In this instance, pacf aims to identify a correlation between time and performance parameters, in turn suggesting if a specific pattern of column types was manufactured over time. Additional details on acf and pacf are also provided in the SI.

The *h* and *A*_s_ time series were first resampled by averaging the data set in day intervals, irrespective of the other column parameters. Other than reducing noise, resampling is customary when autocorrelation analysis is executed over large time periods [58, 61].

**FIGURE 1:**
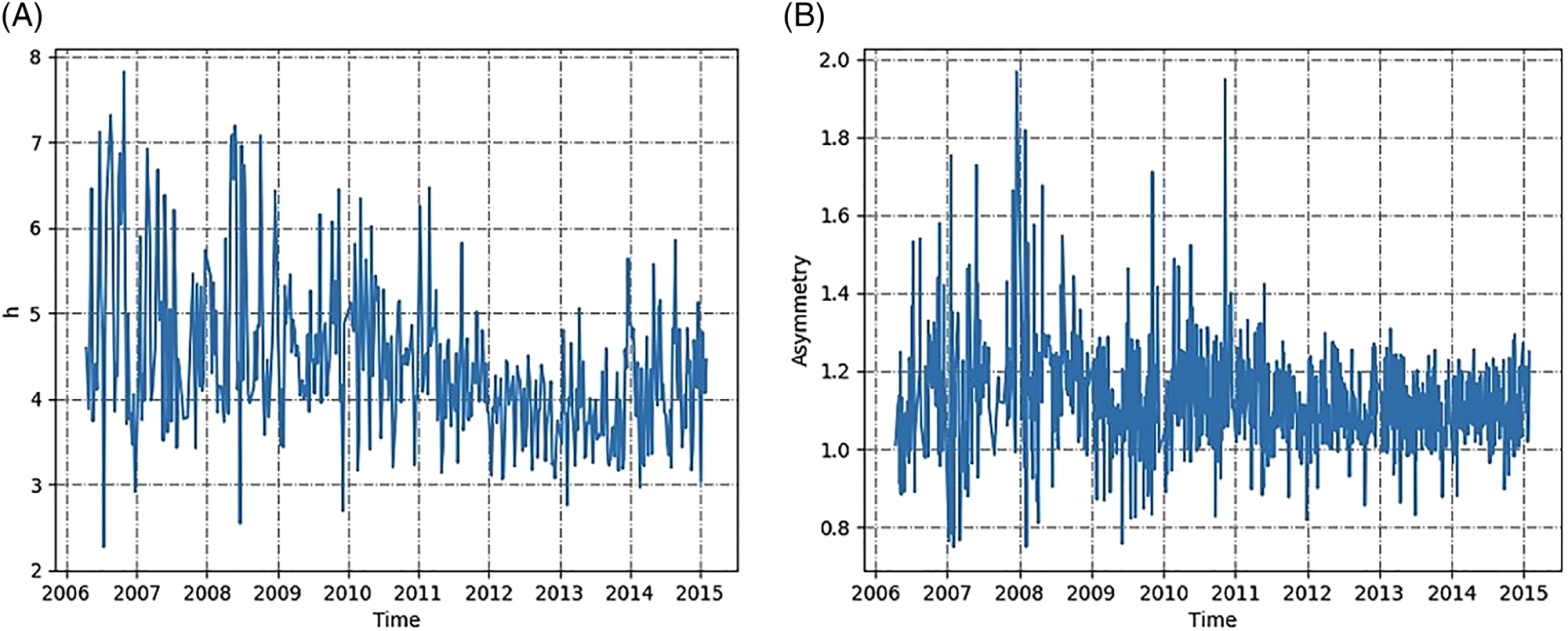
Time series of (A) reduced plate height, h, and (B) asymmetry, $A_{s}$, for pre‐packed purification columns manufactured over the 10‐year period monitored

Figure 1 shows the time series of the two performance parameters, *h* and *A_s_*. Over the 10 year time considered, the h values varied between about 7.8 and 2.2, with an average of around 4.5. Variability reduced significantly from 2011 onward, with a slight decrease of plate height in 2012–2013. The asymmetry ranged between about 2 and 0.8, with an average of 1.1. Similar to plate height, the scatter in the asymmetry over the first 5 years is larger than after 2011. According to Scharl et al. [7], industrial quality assurance tests require a column to have h comprised should be smaller than 5 in industry, while the acceptable range for $A_{s}$ is between 0.8 and 1.6 [7]. The observed variability is a natural consequence of industrial manufacturing, yet the columns produced were within specifications in terms of both h and *A_S_*.

**FIGURE 2:**
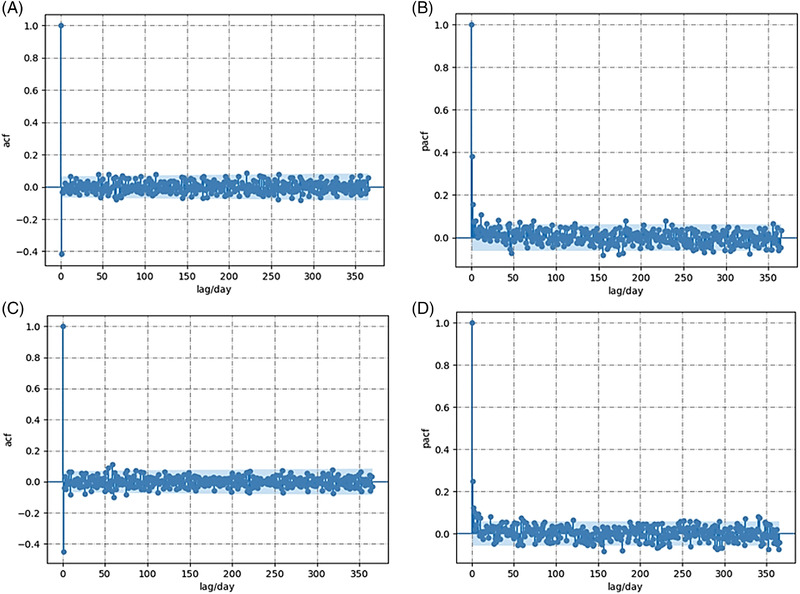
Autocorrelation (acf) and partial autocorrelation (pacf) analysis of reduced plate height, h, and asymmetry, $A_{s}$. (A) Acf of h; (B) pacf of h; (C) acf of $A_{s}$; (D) pacf of $A_{s}$. The blue shaded areas correspond to a 95% confidence interval

Figure 2 shows the results from the autocorrelation and partial autocorrelation analysis on h and $A_{s}$ using lag time of days up to one year. Other lag times were also examined (i.e., weekly, monthly as well as over 2, 3 months) with no significant difference. For both h and $A_{s}$, almost all of the acf and pacf coefficients lie within the 95% confidence interval. The low acf demonstrates that the dataset does not have a specific pattern with time, quantitatively confirming that the manufacturing line was stable over the 10‐year period here investigated [63]. In addition, low pacf rules out the existence of confounding variables such as certain patterns in terms of column sampling and testing over time. In other words, pacf analysis confirms that column manufacture was unbiased, excluding the possibility that a certain column type (for example, having a specific size and packed with a specific particle) was manufactured predominantly over other columns over time. Overall, acf and pacf demonstrate that all performance tests were time‐independent, making the data set solely dependent on the five input parameters of particle size, column diameter, column length, column backbone, and functional mode.

### 3.2 Influence of column parameters on packing quality

XGBoost was utilized to assess the influence of the column parameters (i.e., the inputs to ML algorithm: particle size, column length, column diameter, functional mode, and resin backbone) on packing quality (i.e., ML outputs of h and $A_{s}$). Other ML algorithms such as artificial neural networks and decision‐tree were also employed in a preliminary model assessment (refer to SI for additional information on ML models). XGBoost consistently provided the highest predictive precision, mainly due to its regularization and shrinkage terms (Equations (2) and (3)) being capable of curbing over‐fitting, the main cause of poor prediction.

**FIGURE 3:**
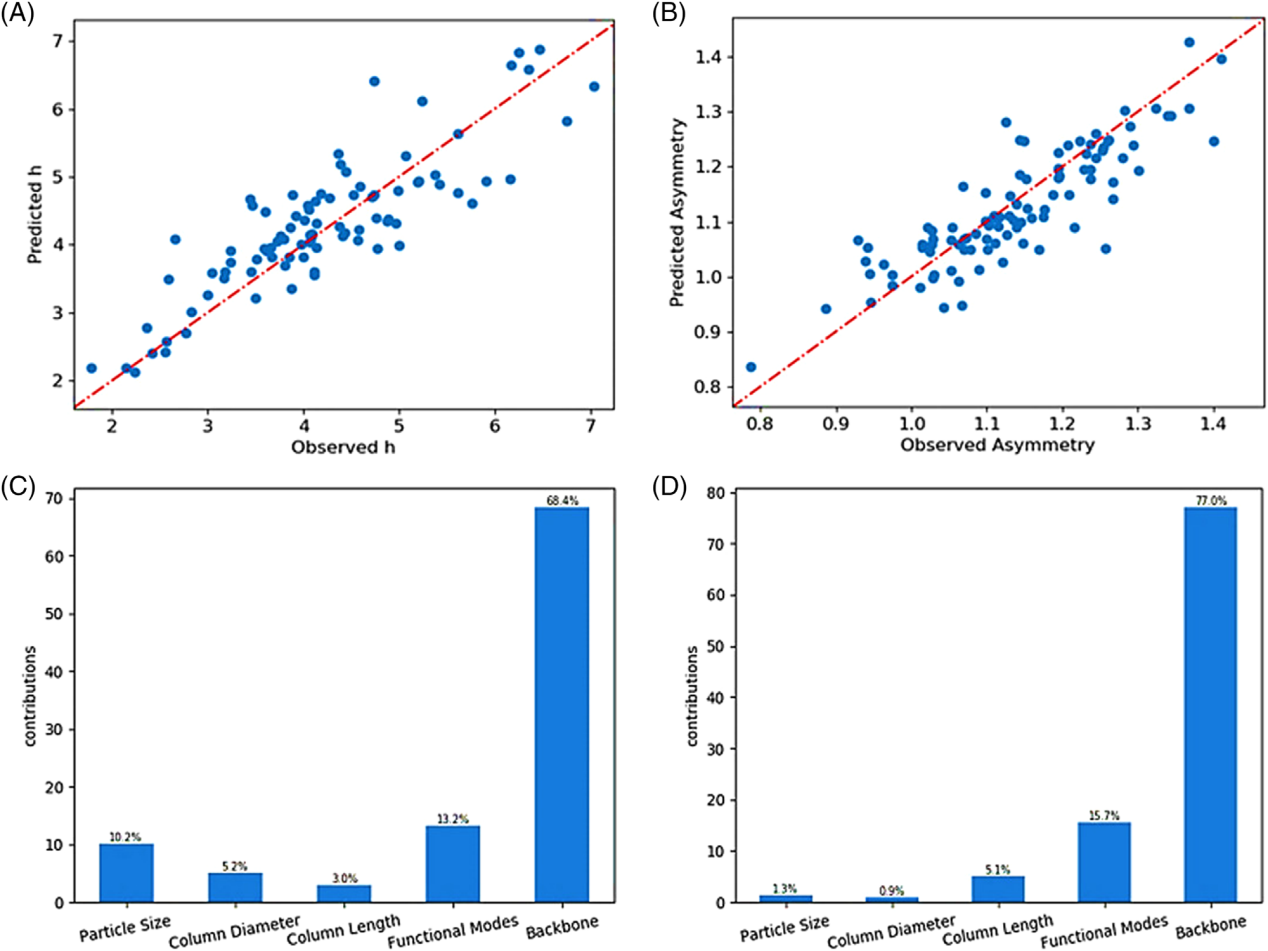
XGBoost prediction results for (A) h and (B) $A_{s}$ over the testing data set. Variable importance contributions of (C) h and (D) $A_{s}$ are reported. The importance is calculated based on the improvement of the performance measured by each attribute split point, weighted by the number of observations the node is responsible for. The importance contributions, named by Gain in XGBoost (refer to Equations (4) and (5)), were transferred into a percentage

**FIGURE 4:**
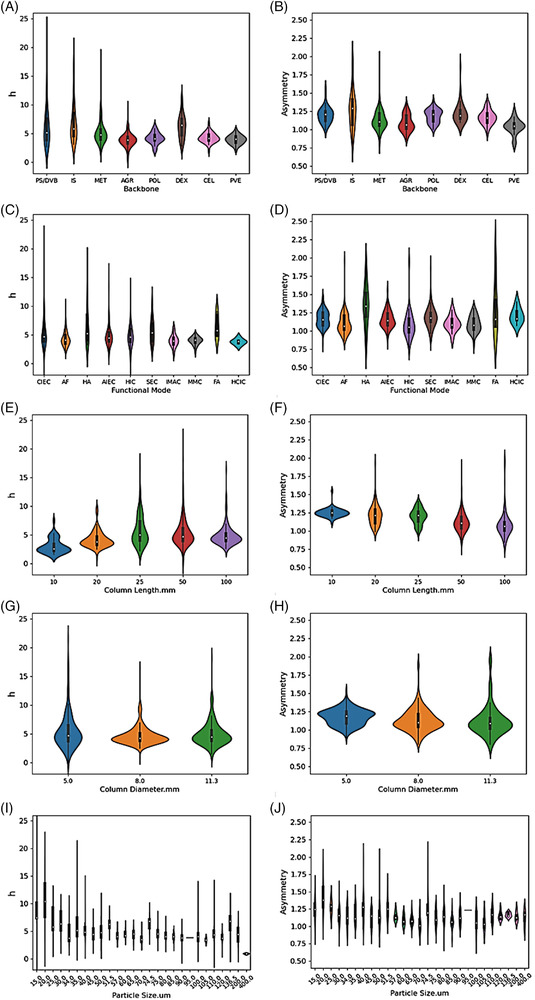
Violin plots of h and *A_s_* against input parameters (backbone, functional mode, column length, column diameter, particle diameter). (A) h versus backbone (PS/DVB: polystyrene divinylbenzene; IS: Inorganic support; MET: Methacrylate; AGR: Agarose; POL: Polymer grafted; DEX: Dextran; CEL: Cellulose; PVE: polyvinyl‐ether hydrophilic). (B) $A_{s}$ versus backbone. (C) h versus functional mode (CIEC: cation exchange chromatography; AF: affinity chromatography; HA: hydroxyl‐apatite chromatography; AIEC: anion exchange chromatography; HIC: hydrophobic interaction chromatography; SEC: size‐exclusion chromatography; IMAC: immobilized metal affinity chromatography; MMC: mixed‐mode chromatography; FA: fluorophore adsorption chromatography; HCIC: hydrophobic charge induction chromatography). (D) $A_{s}$ versus functional mode. (E) h versus column length. (F) $A_{s}$ versus column length. (G) h versus column diameter. (H) $A_{s}$ versus column diameter. (I) h versus particle diameter. (J) $A_{s}$ versus particle diameter

Figure 3 summarizes the results obtained with the XGBoost model to predict the experimental data. In particular, Figure 3a,b compares the predicted h and $A_{s}$, respectively, against the observed data of the testing data set. The predictions are in good agreement with the experimental results, where the MAPE of predicted results to the observed values are 10% for h and 7% for $A_{s}$, with a few outliers in the 40%–50% range. These acceptable errors confirm that the XGBoost model can be applied to this problem with good prediction accuracy. Figure 3c,d reports the contribution importance, I (Equations (4) and (5)), of the various input parameters to predict the model outputs. Interestingly, column backbone resulted as the most important descriptor of packing quality, accounting for 68.4% and 77.0% for the prediction of h and $A_{s}$, respectively. Functional mode was the second most significant descriptor for the estimation of packing quality, accounting for about 15% contribution importance, followed in various order by the other parameters (particle size, column diameter, and column length). Violin plots were employed to further analyze the correlation between input features and column performance (Figure 4). A violin plot is an extension of a box and whisker plot, clearly recognizable inside the “violins”, decorated with a curve whose width is related to the probability density.

#### 3.2.1 Resin backbone

Resin backbone was the most influential parameter for the prediction of packing quality. The material making up the resin backbone can be either inorganic, synthetic polymer, or natural polymer. The nature of the material employed determines the number of properties such as surface roughness of the particles [64], particle size distribution (linked to the manufacturing method)[65], the occurrence of microstructural defects, and other mechanical properties such as Young modulus and density [63, 64]. All these factors impact column packing, either directly or indirectly, in turn influencing the homogeneity of the resulting chromatographic bed, that is, packing quality. Johnson et al. examined a range of resin materials (agarose, cellulose, ceramic) through X‐ray computed tomography (CT) and focused ion beam [66]. They highlighted clear variations in the chemical, physical and mechanical properties of the different materials. Our analysis with the XGBoost model also confirms that resin characteristics strongly influence chromatographic performance.

Figure 4a,b presents violin plots of *h* and *A_s_*, respectively, over the eight different backbones tested. It is possible to observe that certain backbones have worse performance than others as measured by both of the two packing quality parameters *h* and *A_s_*. For example, polystyrene‐divinylbenzene, inorganic support (IS) and dextran have data widely distributed, with an average *h* above 5 and an average *A_s_* above 1.2. On the other hand, agarose, cellulose, and PVE hydrophilic (PVE) demonstrated consistent results (little data scatter) with average *h* and *A_s_* well below the arbitrary thresholds of 5 and 1.2, respectively. This analysis clearly demonstrates the importance of backbone selection, for example, during the process or method development.

It is worth noting that IS was relatively popular in the first 3 years of our data set, while PVE hydrophilic (PVE) matrices were little used at first, becoming more mainstream after 2011. This change in backbone population over time can partly explain the slight decrease of the absolute value of *h*, as well as the reduced scatter of h and $A_{s}$ observed from 2011 onward (Figure 1).

#### 3.2.2 Functional mode

The functional mode was the second most important parameter to predict packing quality. Figure 4c,d shows the relation between h and $A_{s}$ over the different functional modes. The influence of the functionalization chemistry on column packing is less intuitive than for chromatographic backbone. Stickel and Fotopoulos [67] reported the difference in the pressure‐flow profiles between sepharose and phenyl sepharose, which was associated with the differing hydrophobic and electrostatic character of the resin beads. Electrostatic and hydrophobic interactions might promote local or temporary bonding of two or more particles into clusters, decreasing the degrees of freedom of the slurry, and thus influencing column packing [68]. Also, functionalization procedures can change the mechanical and surface properties of the beads, for example, as a consequence of the different solvents, chemicals, and temperatures employed for ligand immobilization. This in turn influences the packing process [69], ultimately determining packing quality.

**FIGURE 5:**
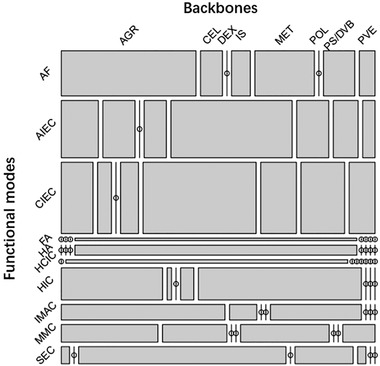
Mosaic plot of the combinations of functional mode and backbone material tested. The size of the tiles represents the relative frequency of each combination. PS/DVB: polystyrene divinylbenzene; IS: Inorganic support; MET: Methacrylate; AGR: Agarose; POL: Polymer grafted; DEX: Dextran; CEL: Cellulose; PVE: polyvinyl‐ether hydrophilic; CIEC: cation exchange chromatography; AF: affinity chromatography; HA: hydroxyl‐apatite chromatography; AIEC: anion exchange chromatography; HIC: hydrophobic interaction chromatography; SEC: size‐exclusion chromatography; IMAC: immobilized metal affinity chromatography; MMC: mixed‐mode chromatography; FA: fluorophore adsorption chromatography; HCIC: hydrophobic charge induction chromatography

The possibility of a correlation between column functionality and backbone was tested both qualitatively (mosaic plot in Figure 5) and statistically by employing the chi‐squared test. The size of the mosaic tiles in Figure 5 is proportional to the number of chromatographic columns in the data set having a certain combination of backbone and functional mode. Some of the tiles are predominant over the others, for example, agarose and methacrylate‐based materials are employed across affinity, ion exchange, and hydrophobic interaction chromatography (AF, AIEC, CIEC, HIC, IMAC, MMC in Figure 5). Such columns are indeed ubiquitous in the downstream processing of biopharmaceuticals. Other backbones find use in specific application domains, for example, dextran is predominantly employed for SEC, and HCIC is purely carried out with cellulosic adsorbents. In addition, a number of combinations of functional mode and backbone are not represented in the data set, indicating some resin materials do not find a use for certain chromatographic modalities. A chi‐squared test of independence with 63 degrees of freedom, that is, (8 backbones – 1) x (10 functional modes – 1), and with a sample size of 546 tests indeed showed a significant relationship between the two input variables, ${\chi_{t}}^{2}\left(63,\mathrm{num}=546\right)=693,p<0.01$. While a correlation between resin material and functionalisation is apparent, its influence in the ML model was eliminated by averaging all experimental results measured under the same input conditions (see Section 2.1), an especially important step to prevent the same samples from being present in both the training and testing set thus overestimating the accuracy.

#### 3.2.3 Column length

The influence of column length on *h* is presented in Figure 4e. It is possible to observe that the median for h, as well as its propensity to data, scatter, and relatively large values (h above 10) increase with column length. This observation can be explained by a combination of packing consolidation and wall effects. The former is relevant during column manufacture, that is when compression forces transfer through the packing via inter‐particle friction as well as friction between particles and the column wall [70]. The uneven stress distribution created between particles in the bulk and at the periphery of the column negatively affects bed consolidation and packing homogeneity. The presence of the wall constrains the resin particles to pack in configurations with higher local porosity in the immediate vicinity of the column wall. The columns investigated in this work were small‐scale purification columns (column volume about 1 and 10 mL) with relatively large particle diameters (15–400 μm) and small column diameters (5–11.3 mm). The resulting column diameter to particle diameter ratio was in general around 80, down to 20 for some columns. In this context, Maier et al. [71] reported wall effect on axial dispersion can be observed even for columns with column diameter to particle diameter ratio greater than 100. Reising et al. [72] and Gritti [73] studied the dependence of fluid velocity with radial position, and concluded that the velocity close to the column wall can be up to 2.2 times the bulk velocity, significantly contributing to band broadening and early breakthrough. Flow non‐idealities arising from both uneven packing difficulties and wall effects scale with column length, with packing quality and column performance inversely related to it.

The contribution of column length on *A_s_* is reported in Figure 4f. No significant difference can be observed across the data, other than a minor decrease in the median asymmetry with column length. Asymmetry is heavily determined by extra column band broadening, that is, related to all flow non‐idealities present in the extra column volumes such as tubing, fitting, column distributor and collectors, pumps, valves, etc. This effect becomes more prominent for smaller columns, as described by Kaltenbrunner et al. [74] who reported extra column volumes accounting for more than 90% band broadening in small columns.

#### 3.2.4 Column diameter

According to ML results, the contribution of column diameter to the prediction of *h* is 5.2%, while it is only 0.9% for $A_{s}$ (Figure 3c), and no clear relationship can be observed between column diameter and the two performance output parameters (Figure 4 g,h). All the three‐column diameters considered in this work fall in the same order of magnitude (5, 8, and 11.3 mm), thus hiding any potential correlation between column diameter and packing quality. Schweiger et al. [3] analyzed the band broadening arising from the extra‐column and in‐column contributions of pre‐packed columns with different column diameters, and concluded that an increase in column diameter can lead to an increase in peak width as caused by flow non‐idealities in the flow distributor and collector. Experimental data for wider columns is required to identify and eventually quantify any possible relationship between column diameter and column performance.

#### 3.2.5 Particle diameter

The correlation between particle diameter and h is reported in Figure 4i. Accordingly, to the reduced form of the van Deemter equation (Equation (8)), the magnitude of h is not dependent on particle diameter. ML results indicate that the important contribution of particle diameter to h is 10.7% (Figure 3c). In Figure 4i the median h slightly drops with particle size, possibly resulting from packing difficulties with smaller particles, as also reported by Scharl et al. [7]. No trend between $A_{s}$ and particle size could be observed (Figure 4j).

## 4 CONCLUDING REMARKS

Traditional statistical analysis (for example, autocorrelation analysis, chi‐square analysis) and ML were applied to a large data set (546 different combinations of column features) of packing quality (reduced plate height, *h*, and asymmetry, *A_s_*) for pre‐packed columns manufactured with different column sizes (column length and column diameter) and packed with different resins (backbone, functional mode, and particle diameter) over 10 years.

Autocorrelation and partial autocorrelation provided a quantitative framework to analyze column quality over time. The results indicate that packing quality was indeed not correlated with time, indicating that column manufacture, sampling, and testing were consistent over the 10 years.

The XGBoost represented an excellent ML model to predict column performance, with MAPE of 10 and 7% on *h* and *A_s_*, respectively. According to the ML tool employed, the column backbone contributed the most to its predictive capability. In other words, the resin material employed had the most significant impact on column performance. A trend between column length and performance was also observed, with h raising slightly as the length increased, consistent with a larger contribution to band broadening due to wall effects and axial dispersion.

Overall, this work demonstrates the capability of ML to evaluate and predict column performance solely from the knowledge of some basic column characteristics (column length and diameter, particle size, backbone material, and functional mode). These results could be employed to extrapolate the expected performance characteristics on new and existing columns types, help set QA protocols for new and existing manufacturing lines for pre‐packed chromatography columns, or as a reference benchmark for columns packed traditionally in lab settings, especially for hard to pack columns such as polystyrene‐divinylbenzene and ISs. The results presented here can guide further efforts in column optimization, for example, informing potential inefficiencies in the packing process, and suggesting improvements of backbone and functional modes to obtain easy to pack resins prone to form ordered packing arrangements with high chromatographic performance.

More in general, ML provides a quantitative tool to describe complex problems with multiple input features, including categorical features such as resin backbone and functional mode. ML methods can also be employed in other chromatographic areas, for example, for generating accurate retention models, resolving complex chromatography peaks, and for searching column structures with improved performance.

## CONFLICT OF INTEREST

 The authors declare that they have no conflict of interest.

## Supporting information

SUPPORTING INFORMATION

## Data Availability

The data that support the findings of this study are available from Repligen. Restrictions apply to the availability of these data, which were used under license for this study. Data are available from Dr. Tim Schroeder or Dr. Theresa Scharl with the permission of Repligen.

## References

[1] Brenac Brochier V , Chabre H , Lautrette A , Ravault V , Couret MN , Didierlaurent A , Moingeon P . High throughput screening of mixed‐mode sorbents and optimisation using pre‐packed lab‐scale columns for the purification of the recombinant allergen rBet v 1a. J Chromatogr B Anal Technol Biomed Life Sci. 2009;877:2420–7. 10.1016/j.jchromb.2009.03.020 19345650

[2] Shukla AA , Gottschalk U . Single‐use disposable technologies for biopharmaceutical manufacturing. Trends Biotechnol. 2013;31:147–54. 23178074 10.1016/j.tibtech.2012.10.004

[3] Schweiger S , Jungbauer A . Scalability of pre‐packed preparative chromatography columns with different diameters and lengths taking into account extra column effects. J Chromatogr A. 2018;1537:66–74. 10.1016/j.chroma.2018.01.022. 29373126

[4] Schweiger S , Hinterberger S , Jungbauer A . Column‐to‐column packing variation of disposable pre‐packed columns for protein chromatography. J Chromatogr A. 2017;1527:70–9. 29089107 10.1016/j.chroma.2017.10.059

[5] Glueckauf E , Glueckauf E . Theory of chromatography. Part 9. The “theoretical plate” concept in column separations. Transactions of the Faraday Society 1955;51:34–44.

[6] Kaltenbrunner O , Watler P , Yamamoto S . Column qualification in process ion‐exchange chromatography. Progress in Biotechnology 2000;16(C):201–206.

[7] Scharl T , Jungreuthmayer C , Dürauer A , Schweiger S , Schröder T , Jungbauer A . Trend analysis of performance parameters of pre‐packed columns for protein chromatography over a time span of ten years. J Chromatogr A. 2016;1465:63–70. 27575920 10.1016/j.chroma.2016.07.054

[8] Fornstedt T , Zhong G , Guiochon G . Peak tailing and mass transfer kinetics in linear chromatography. J Chromatogr A. 1996;741:1–12.

[9] Fornstedt T , Zhong G , Guiochon G . Peak tailing and slow mass transfer kinetics in nonlinear chromatography. J Chromatogr A. 1996;742:55–68.

[10] Wakamatsu A , Morimoto K , Shimizu M , Kudoh S . A severe peak tailing of phosphate compounds caused by interaction with stainless steel used for liquid chromatography and electrospray mass spectrometry. J Sep Sci. 2005;28:1823–30. 16224979 10.1002/jssc.200400027

[11] Kirkland JJ , Yau WW , Stoklosa HJ , Dilks CH . Sampling and extra‐column effects in high‐performance liquid chromatography; influence of peak skew on plate count calculations. J Chromatogr Sci. 1977;15:303–16. 10.1093/chromsci/15.8.303. 893648

[12] Chapel S , Heinisch S . Strategies to circumvent the solvent strength mismatch problem in online comprehensive two‐dimensional liquid chromatography. J Sep Sci. 2022;45:7–26. 34525266 10.1002/jssc.202100534

[13] Mitchell NS , Hagel L , Fernandez EJ . In situ analysis of protein chromotography and column efficiency using magnetic resonance imaging. J Chromatogr A. 1997;779:73–89. 10.1016/S0021-9673(97)00457-3. 9335119

[14] Carta G , Jungbauer A . Protein chromatography: process development and scale‐up. John Wiley & Sons; 2020 Jun 2.

[15] Guiochon G , Felinger A , Shirazi DG . Fundamentals of preparative and nonlinear chromatography. Amsterdam, NL: Elsevier; 2006. 10.1016/j.chroma.2016.03.050 27079748

[16] Dimartino S , Boi C , Sarti GC . A validated model for the simulation of protein purification through affinity membrane chromatography. J Chromatogr A. 2011;1218:1677–90. 21168846 10.1016/j.chroma.2010.11.056

[17] Dimartino S , Boi C , Sarti GC . Scale‐up of affinity membrane modules: comparison between lumped and physical models. J Mol Recognit. 2015;28:180–90. 25663188 10.1002/jmr.2406

[18] Neal RM . Pattern recognition and machine learning. Technometrics 2007;49. 10.1198/tech.2007.s518.

[19] Toth CD , O'Rourke J , Goodman JE (eds). Handbook of discrete and computational geometry. 3rd ed. Boca Raton, FL: CRC Press LLC; 2017.

[20] Rasouli K , Hsieh WW , Cannon AJ . Daily streamflow forecasting by machine learning methods with weather and climate inputs. J Hydrol. 2012;414–415:284–93.

[21] Xu X , Zhang Y , Zou L , Wang M & Li A A gene signature for breast cancer prognosis using support vector machine. 2012 5th Int Conf Biomed Eng Informatics: BMEI 2012 2012:928–31.

[22] Bos TS , Knol WC , Molenaar SRA , Niezen LE , Schoenmakers PJ , Somsen GW , Pirok BWJ . Recent applications of chemometrics in one‐ and two‐dimensional chromatography. J Sep Sci. 2020;43:1678–727. 32096604 10.1002/jssc.202000011 PMC7317490

[23] Risum AB , Bro R . Using deep learning to evaluate peaks in chromatographic data. Talanta 2019;204:255–60. 31357290 10.1016/j.talanta.2019.05.053

[24] Kantz ED , Tiwari S , Watrous JD , Cheng S , Jain M . Deep neural networks for classification of LC‐MS spectral peaks. Anal Chem. 2019;91:12407–13. 31483992 10.1021/acs.analchem.9b02983 PMC7089603

[25] Marengo E , Gianotti V , Angioi S , Gennaro MC . Optimization by experimental design and artificial neural networks of the ion‐interaction reversed‐phase liquid chromatographic separation of twenty cosmetic preservatives. J Chromatogr A. 2004;1029:57–65. 15032350 10.1016/j.chroma.2003.12.044

[26] Hervás C , Martínez AC , Silva M , Serrano JM . Improving the quantification of highly overlapping chromatographic peaks by using product unit neural networks modeled by an evolutionary algorithm. J Chem Inf Model. 2005;45:894–903. 16045283 10.1021/ci049697o

[27] Vasiljević T , Onjia A , Čokeša D , Laušević M . Optimization of artificial neural network for retention modeling in high‐performance liquid chromatography. Talanta 2004;64:785–90. 18969673 10.1016/j.talanta.2004.03.032

[28] Kensert A , Collaerts G , Efthymiadis K , Desmet G , Cabooter D . Deep Q‐learning for the selection of optimal isocratic scouting runs in liquid chromatography. J Chromatogr A. 2021;1638:461900. 33485027 10.1016/j.chroma.2021.461900

[29] Ben Hameda A , Elosta S , Havel J . Optimization of the capillary zone electrophoresis method for Huperzine A determination using experimental design and artificial neural networks. J Chromatogr A. 2005;1084:7–12. 16114229 10.1016/j.chroma.2004.10.097

[30] Wang G , Briskot T , Hahn T , Baumann P , Hubbuch J . Estimation of adsorption isotherm and mass transfer parameters in protein chromatography using artificial neural networks. J Chromatogr A. 2017;1487:211–7. 28159368 10.1016/j.chroma.2017.01.068

[31] Narayanan H , Seidler T , Luna MF , Sokolov M , Morbidelli M , Butté A . Hybrid Models for the simulation and prediction of chromatographic processes for protein capture. J Chromatogr A. 2021;1650:462248. 34087519 10.1016/j.chroma.2021.462248

[32] Narayanan H , Luna MF , Stosch M , Cruz Bournazou MN , Polotti G , Morbidelli M , Butté A , Sokolov M . Bioprocessing in the digital age: the role of process models. Biotechnol J. 2020;15:1900172. 10.1002/biot.201900172 31486583

[33] Narayanan H , Behle L , Luna MF , Sokolov M , Guillén‐Gosálbez G , Morbidelli M , Butté A . Hybrid‐EKF: hybrid model coupled with extended Kalman filter for real‐time monitoring and control of mammalian cell culture. Biotechnol Bioeng. 2020;117:2703–14. 32436988 10.1002/bit.27437

[34] Cussler EL , Cussler EL . Diffusion: mass transfer in fluid systems. 3rd ed. Cambridge, UK: Cambridge University Press; 2009.

[35] Yeh HS , Wills GB . Diffusion coefficient of sodium nitrate in aqueous solution at 25.deg. as a function of concentration from 0.1 to 1.0M. J Chem Eng Data. 1970;15:187–9.

[36] Chen T , Guestrin C . XGBoost: a scalable tree boosting system. Proc ACM SIGKDD Int Conf Knowl Discov Data Min .13–17 Aug 2016: 785–94.

[37] Freund Y , Mason L . The alternating decision tree learning algorithm. Int Conf Mach Learn . 1999. 10.1093/jxb/ern164.

[38] Lewis RJ , Street WC . An introduction to classification and regression tree (CART) analysis. 2000 Ann Meet Soc Acad Emerg Med . 2000. ht tps://doi.org/10.1.1.95.4103.

[39] Friedman JH . Greedy function approximation: a gradient boosting machine. Ann Stat. 2001;29:1189–232.

[40] Knapp SK . Accelerate FPGA macros with one‐hot approach. Electron Design 1990.

[41] Singh D , Singh B . Investigating the impact of data normalization on classification performance. Applied Soft Computing. 2020;97:105524.

[42] Pedregosa F , Varoquaux G , Gramfort A , Michel V , Thirion B , Grisel O , Blondel M , Prettenhofer P , Weiss R , Dubourg V , Vanderplas J , Passos A , Cournapeau D , Brucher M , Perrot M , Duchesnay É . Scikit‐learn: machine learning in Python. The Journal of machine Learning research. 2011.

[43] Chen T , He T , Benesty M , Khotilovich V . Package ‘xgboost’. 2019.

[44] Chai T , Draxler RR . Root mean square error (RMSE) or mean absolute error (MAE)? ‐Arguments against avoiding RMSE in the literature. Geosci Model Dev. 2014;7:1247–50.

[45] Wilson EJ , Geankoplis CJ . Liquid mass transfer at very low reynolds numbers in packed beds. Ind Eng Chem Fundam. 1966;5:9–14.

[46] Sarwar MS , Simon U , Dimartino S . Experimental investigation and mass transfer modelling of 3D printed monolithic cation exchangers. J Chromatogr A. 2021;1646:462125. 33894456 10.1016/j.chroma.2021.462125

[47] Guiochon G , Gritti F . Shell particles, trials, tribulations and triumphs. J Chromatogr A. 2011;1218:1915–38. 21353228 10.1016/j.chroma.2011.01.080

[48] Gritti F , Leonardis I , Abia J , Guiochon G . Physical properties and structure of fine core–shell particles used as packing materials for chromatography. J Chromatogr A. 2010;1217:3819–43. 20447642 10.1016/j.chroma.2010.04.026

[49] Knox JH . Band dispersion in chromatography ‐ A universal expression for the contribution from the mobile zone. J Chromatogr A. 2002;960:7–18. 12150565 10.1016/s0021-9673(02)00240-6

[50] Patel KD , Jerkovich AD , Link JC , Jorgenson JW . In‐depth characterization of slurry packed capillary columns with 1.0‐μm nonporous particles using reversed‐phase isocratic ultrahigh‐pressure liquid chromatography. Anal Chem. 2004;76:5777–86. 15456298 10.1021/ac049756x

[51] Malkin DS , Wei B , Fogiel AJ , Staats SL , Wirth MJ . Submicrometer plate heights for capillaries packed with silica colloidal crystals. Anal Chem. 2010;82:2175–7. 20158216 10.1021/ac100062t PMC3018733

[52] Khirevich S , Daneyko A , Höltzel A , Seidel‐Morgenstern A , Tallarek U . Statistical analysis of packed beds, the origin of short‐range disorder, and its impact on eddy dispersion. J Chromatogr A. 2010;1217:4713–22. 20570271 10.1016/j.chroma.2010.05.019

[53] Gritti F , Leonardis I , Shock D , Stevenson P , Shalliker A , Guiochon G . Performance of columns packed with the new shell particles, Kinetex‐C18. J Chromatogr A. 2010;1217:1604–15. 10.1016/j.chroma.2009.12.079. 20116065

[54] Dolamore F , Dimartino S , Fee CJ . Numerical elucidation of flow and dispersion in ordered packed beds: nonspherical polygons and the effect of particle overlap on chromatographic performance. Anal Chem. 2019;91:15009–16. 31684719 10.1021/acs.analchem.9b03598

[55] Knox JH . Band dispersion in chromatography ‐ a new view of A‐term dispersion. J Chromatogr A. 1999;831:3–15. 10.1016/s0021-9673(02)00240-6 12150565

[56] Lan T , Gerontas S , Smith GR , Langdon J , Ward JM , Titchener‐Hooker NJ . Investigating the use of column inserts to achieve better chromatographic bed support. Biotechnol Prog. 2012;28:1285–91. 22753390 10.1002/btpr.1597

[57] Jaulmes A , Ignatiadis I , Cardot P , Vidal‐Madjar C . Characterization of peak asymmetry with overloaded capillary columns. J Chromatogr A. 1987;395:291–306.

[58] Pápai Z , Pap TL . Analysis of peak asymmetry in chromatography. J Chromatogr A. 2002;953:31–8. 12058945 10.1016/s0021-9673(02)00121-8

[59] Miyabe K , Guiochon G . Estimation of the column radial heterogeneity from an analysis of the characteristics of tailing peaks in linear chromatography. J Chromatogr A. 1999;830:29–39. 10.1016/s0021-9673(99)00752-9 10536826

[60] Miyabe K , Guiochon G . Peak tailing and column radial heterogeneity in linear chromatography. J Chromatogr A. 1999;830:263–74. 10.1016/s0021-9673(99)00752-9 10536826

[61] Madsen H . Time series analysis. Chapman and Hall/CRC; 2007.

[62] Palma W . Long‐memory time series. Hoboken, NJ: John Wiley & Sons, Inc.; 2007.

[63] Mills TC . ARMA models for stationary time series. Appl Time Ser Anal. 2019;31–56.

[64] Bendada K , Hamdi B , Boudriche L , Balard H , Calvet R . Surface characterization of reservoir rocks by inverse gas chromatography: effect of a surfactant. Colloids Surfaces A Physicochem Eng Asp. 2016;504:75–85.

[65] Bacskay I , Sepsey A , Felinger A . Determination of the pore size distribution of high‐performance liquid chromatography stationary phases via inverse size exclusion chromatography. J Chromatogr A. 2014;1339:110–7. 24666937 10.1016/j.chroma.2014.02.085

[66] Johnson TF , Bailey JJ , Iacoviello F , Welsh JH , Levison PR , Shearing PR , Bracewell DG . Three dimensional characterisation of chromatography bead internal structure using X‐ray computed tomography and focused ion beam microscopy. J Chromatogr A. 2018;1566:79–88. 29970222 10.1016/j.chroma.2018.06.054

[67] Stickel JJ , Fotopoulos A . Pressure‐flow relationships for packed beds of compressible chromatography media at laboratory and production scale. Biotechnol Prog. 2001;17:744–51. 11485438 10.1021/bp010060o

[68] Kawachi Y , Ikegami T , Takubo H , Ikegami Y , Miyamoto M , Tanaka N . Chromatographic characterization of hydrophilic interaction liquid chromatography stationary phases: Hydrophilicity, charge effects, structural selectivity, and separation efficiency. J Chromatogr A. 2011;1218:5903–19. 21782195 10.1016/j.chroma.2011.06.048

[69] McCue JT , Cecchini D , Hawkins K , Dolinski E . Use of an alternative scale‐down approach to predict and extend hydroxyapatite column lifetimes. J Chromatogr A. 2007;1165:78–85. 10.1016/j.chroma.2007.07.053. 17706660

[70] Dorn M , Eschbach F , Hekmat D , Weuster‐Botz D . Influence of different packing methods on the hydrodynamic stability of chromatography columns. J Chromatogr A. 2017;1516:89–101. 28818329 10.1016/j.chroma.2017.08.019

[71] Maier RS , Kroll DM , Davis HT . Diameter‐dependent dispersion in packed cylinders. AIChE J. 2007;53:527–30.

[72] Reising AE , Schlabach S , Baranau V , Stoeckel D , Tallarek U . Analysis of packing microstructure and wall effects in a narrow‐bore ultrahigh pressure liquid chromatography column using focused ion‐beam scanning electron microscopy. J Chromatogr A. 2017;1513:172–82. 28739273 10.1016/j.chroma.2017.07.049

[73] Gritti F . On the relationship between radial structure heterogeneities and efficiency of chromatographic columns. J Chromatogr A. 2018;1533:112–26. 29254865 10.1016/j.chroma.2017.12.030

[74] Kaltenbrunner O , Jungbauer A , Yamamoto S . Prediction of the preparative chromatography performance with a very small column. J Chromatogr A. 1997;760:41–53.

