# Supplementary material for: Prediction of the performance of pre‐packed purification columns through machine learning
Source: J Sep Sci. 2022 Mar 20;45(8):1445–57. doi: 10.1002/jssc.202100864 (PMC9310636; doi:10.1002/jssc.202100864)
Supplement: Supplementary file 1 — SUPPORTING INFORMATION [file JSSC-45-1445-s001.docx]

# Supporting Information

1. **Data Average Process**

We received 24,951 quality control runs of pre-packed small-scale columns over a period of about 10 years. 2232 experimental runs (approximately 10%) were removed from the original data set as they lacked one or more column parameter inputs, reducing the data set to a total of 22,359 tests. Columns with same attributes were manufactured and tested more than once over the ten year monitored, with some popular types examined hundreds of times. Three popular examples are reported in Table 1. The standard error for $h$ and $A_{S}$ was always lower than 10%, indicating that the average $h$ and $A_{S}$ are representative output indicators of column performance for any given column type. After the averaging process, the data set contained a total of 546 independent runs.

| Column Type | Number of tested | Avg $h^{*}$ | Rel err $h$ | Avg $A_{s}^{*}$ | Rel err $A_{s}$ |
| --- | --- | --- | --- | --- | --- |
| 1 | 945 | $3.85\pm0.34$ | 8.8% | $1.14\pm0.06$ | 5.3% |
| 2 | 921 | $7.03\pm0.06$ | 0.8% | $1.26\pm0.06$ | 4.8% |
| 3 | 599 | $4.71\pm0.39$ | 8.3% | $1.03\pm0.05$ | 4.9% |

Table 1. Three popular types of the tested columns. The averaged value (Avg) and standard derivation (Std) of $h$ and $A_{s}$ are reported. Type 1: particle size 90 μm, column diameter 5 mm, column length 50 mm, functional mode HCIC, backbone cellulose; Type 2: particle size 50 μm, column diameter 5 mm, column length 50 mm, functional mode AIEC, backbone inorganic support; Type 3: particle size 65 μm, column diameter 8 mm, column length 100 mm, functional mode CIEC, backbone Methacrylate.

$*$ ± standard deviation.

1. **Introduction of Machine Learning**

The concept of machine learning was firstly proposed by Arthur Samuel in 1959, who is pioneer of artificial intelligence and computer gaming [1]. The early version of machine learning focused on the pattern classification [2–4]. With the continuous improvement of computer computing power, current machine learning focuses on research in the environment of big data [5]. This research mainly focuses on how to effectively use information and obtaining hidden knowledge and patterns from huge amounts of data.

According to the difference in the input features and output, machine learning can be generally distinguished into three basic paradigms: supervised learning, un-supervised learning, and reinforcement learning.

Supervised learning algorithms is used to construct models analyzing data set with specific input and desired outputs [6–8]. The data set here is separated into training data set and testing data set, and each training example in the training data set has one or more inputs and desired outputs. In the mathematical model, the training data set is represented by the matrix. The algorithm iteratively makes predictions on the training data during the training and the parameters in the model are corrected by the supervision functions. Supervised learning algorithms can finally provide a model that can be used to predict output by new outputs within acceptable errors.

The types of the supervised learning include classification and regression based on the problems to solve [7]. When the desired output is restricted to a set of values such as identification of human faces or allocation of items by their features, the classification algorithms are used. The regression algorithms are chosen while the outputs can be any numerical values within a specific range. In this work, the prediction of the column performance belongs to regression problem.

Rather than operating the human-labelled data as supervised learning, the un-supervised learning algorithms can learn from the data set without labels, classifications or categories [9–11]. Instead of providing an output, the un-supervised learning algorithms will identify commonalities in the data and react based on the presence or absence of such commonalities in each new piece of data. So, the un-supervised learning algorithms are widely used in the density estimation in statistics like searching for probability density function.

Reinforcement learning (RL) is used to describe and solve problems that the agent systems learn and interact with the external environment to maximize the return or achieve a specific goal [12–14]. The created agent faces a game-like situation. It solves the problem through trial and error. In order for the agent to act according to the programmer's wishes, agent will be rewarded or punished for the operations it performs. Its goal is to maximize the total reward.

- 1. **Regression Algorithms**

The prediction of column purification performance is based on the regression algorithms. In this section, several famous regression models are introduced.

**Traditional Regression Models**

Linear regression is a basic regression model in machine learning, which assumes that the relationship between input values and targets is linear [15,16]. The linear regression models with one input features are named as single regression, and models with several input features are named as multi-variable regression. This simple model is usually utilized to analyse the clear correlations or combine with other more complex models in ML.

Similar to the linear regression, the polynomial regression is usually applied to fit a curve or a series of curves [17]. Polynomial models are prone to overfitting, so it is important to remove outliers which can distort the prediction curve.

Logistic regression is suitable for the binary dependent variables [18,19]. The logistic model can transfer a continuous variable into binary values, which are widely adopted in classification problems in ML.

**Artificial Neural Network (ANN)**

Because most of the traditional regression models cannot fit the data set at hand perfectly, artificial neural network models will provide more complex models to resolve the prediction problems [20–22]. ANN models are connected by the simple neurons (Figure 2.1), in which are able to make simple mathematical decisions. During solving complex problems, the neurons will work together to provide predictions. An input layer, a hidden layer and an output layer are required to construct a shallow ANN model. The neurons in the hidden layer will transfer the sum of products of inputs with weights and the products of biases with weights into an output signal by the activation function [23] (eg. Sigmoid activation function). The complex models will contain more hidden layers, which will both increase the predictions accuracy and computing power required.


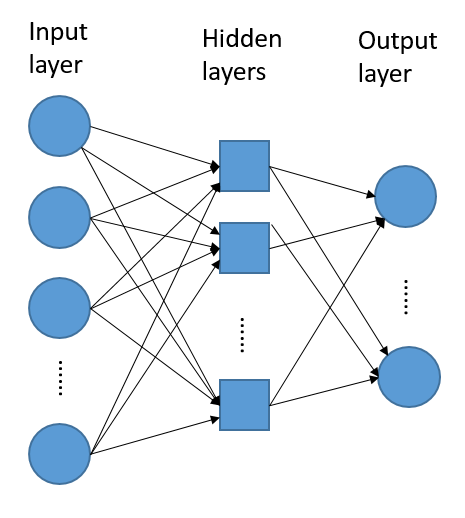


Figure 2.1 A sketch of the shallow ANN model, the blue elements are the neurons connected with each other.

**Decision Tree model**

Decision tree model are one of the most popular models in ML due to their intelligibility and simplicity [24,25]. They can be applied in both classification and regression problems. For a training process of the decision tree, the train data set is split into several subsets (tree leaves) according to a specific index. This splitting process is repeated recursively in the generated subsets, known as recursive segmentation. The recursion stops when the subsets are small enough to have single labels.

There are several types of decision tree model such as boosting trees and rotation forest. Boosting tree is to combine multiple decision trees to enhance the prediction accuracy [26]. Rotation forest allows to adopt principal component analysis (PCA) for the training data set before the training to enhance the performance [27].

Extreme gradient boosting (XGBoost) utilized in this work is based on the decision tree model. The key ideas and mathematical derivation of XGBoost can be found in the main text.

- 1. **Overfitting in Machine Learning**

In statistics, a fit refers to how well the model approximate a target function. In machine learning, regression algorithms seek to approximate the unknown underlying mapping function for the output variables given the input variables.

Overfitting in machine learning means that a model focus on the noises and details in training data set [28,29]. Noises or random fluctuations are treated as concepts in the model, which will not apply for the new data and lead to performance loss. To prevent overfitting, some methods were proposed such as cross-validation [30] and regularization [31], which were utilized in this model.

Cross-validation refers to separate the training data set into multiple mini train-test splits [32]. For a standard k-fold cross-validation, the whole data set is divided into k subsets, which are also known as folds. Then the algorithm is iteratively trained on the k-1 folds subset, and the remaining data set is adopted as test data set. This method can also be adopted for determination of hyper-parameters in the ML model.

Regularization refers to a broad range of techniques for artificially penalizing the complexity of the model. The method of regularization differs in algorithms. For example, trees in decision tree model can be pruned. In ANN algorithm, a dropout layer is involved to simplify the model [33]. In XGBoost, a penalty function is added into regression, which is introduced as Eq 2 and Eq 3 in the main text.

1. **Introduction of ACF and PACF Plot**

Autocorrelation refers to the correlation between the two observations in a time series [34]. When the correlations such as strong positive and negative correlations are present, the current values in time series are influenced by the previous data points. Analyzing the autocorrelation can benefit to understand the properties of time series, and make forecasts based on the known values.

The ACF plot measures and plots the average correlation between data points in a time series and previous values of the series measured for different lag lengths [35]. For example, the correlation for the first lag is measured as the correlation between the time series values measured at time t and all series values measured at time t-1. The correlation for the second lag measures the correlation between the time series values measured at time t and all series values measured at time t-2. PACF plot is similar to an ACF except that each correlation controls for any correlation between observations of a shorter lag length [36]. Thus, the value for the ACF and the PACF at the first lag are same, as both of them measure the correlation between time t data points with time t-1 data points. However, for the second lag, the PACF measures the correlation between time t data points with time t-2 data points after controlling for the correlation between time t data points with data points at time t-1. In this work, ACF and PACF were applied to investigate packing quality changing during the ten year fabrication process.

**References:**

[1] Samuel, A. L., Some studies in machine learning using the game of checkers. *IBM J. Res. Dev.* 1959, 3, 210–229.

[2] Friedman, J. H., Data Mining and Statistics: What’s the connection? *Comput. Sci. Stat.* 1998, 29, 3–9.

[3] Nilsson, N. J., Learning machines. 1965.

[4] Duda, R. O., Hart, P. E., A Wiley-Interscience Publication. 1973.

[5] Jordan, M. I., Mitchell, T. M., Machine learning: Trends, perspectives, and prospects. *Science (80-. ).* 2015, 349, 255–260.

[6] Cunningham, P., Cord, M., Delany, S. J., Machine Learning Techniques for Multimedia. Springer 2008, pp. 21–49.

[7] Hastie, T., Tibshirani, R., Friedman, J., The Elements of Statistical Learning. Springer 2009, pp. 9–41.

[8] Niculescu-Mizil, A., Caruana, R., Proceedings of the 22nd International Conference on Machine Learning. 2005, pp. 625–632.

[9] Barlow, H. B., Unsupervised learning. *Neural Comput.* 1989, 1, 295–311.

[10] Hastie, T., Tibshirani, R., Friedman, J., The Elements of Statistical Learning. Springer 2009, pp. 485–585.

[11] Celebi, M. E., Aydin, K., Unsupervised Learning Algorithms. Springer 2016.

[12] Sutton, R. S., Barto, A. G., Reinforcement Learning: An Introduction. MIT Press 2018.

[13] Kaelbling, L. P., Littman, M. L., Moore, A. W., Reinforcement learning: A survey. *J. Artif. Intell. Res.* 1996, 4, 237–285.

[14] Szepesvári, C., Algorithms for reinforcement learning. *Synth. Lect. Artif. Intell. Mach. Learn.* 2010, 4, 1–103.

[15] Seber, G. A. F., Lee, A. J., Linear Regression Analysis. John Wiley & Sons 2012.

[16] Montgomery, D. C., Peck, E. A., Vining, G. G., Introduction to Linear Regression Analysis. John Wiley & Sons 2021.

[17] Ostertagová, E., Modelling using polynomial regression. *Procedia Eng.* 2012, 48, 500–506.

[18] Menard, S., Applied Logistic Regression Analysis. Sage 2002.

[19] Wright, R. E., Logistic regression. 1995.

[20] Abiodun, O. I., Jantan, A., Omolara, A. E., Dada, K. V., Mohamed, N. A., Arshad, H., State-of-the-art in artificial neural network applications: A survey. *Heliyon* 2018, 4, e00938.

[21] Peterson, C., Rögnvaldsson, T., Lönnblad, L., JETNET 3.0—A versatile artificial neural network package. *Comput. Phys. Commun.* 1994, 81, 185–220.

[22] Hill, T., Marquez, L., O’Connor, M., Remus, W., Artificial neural network models for forecasting and decision making. *Int. J. Forecast.* 1994, 10, 5–15.

[23] Sharma, S., Sharma, S., Athaiya, A., Activation functions in neural networks. *Towar. data Sci.* 2017, 6, 310–316.

[24] Song, Y.-Y., Ying, L. U., Decision tree methods: applications for classification and prediction. *Shanghai Arch. psychiatry* 2015, 27, 130.

[25] Myles, A. J., Feudale, R. N., Liu, Y., Woody, N. A., Brown, S. D., An introduction to decision tree modeling. *J. Chemom. A J. Chemom. Soc.* 2004, 18, 275–285.

[26] Ke, G., Meng, Q., Finley, T., Wang, T., Chen, W., Ma, W., Ye, Q., Liu, T.-Y., Lightgbm: A highly efficient gradient boosting decision tree. *Adv. Neural Inf. Process. Syst.* 2017, 30, 3146–3154.

[27] Rodriguez, J. J., Kuncheva, L. I., Alonso, C. J., Rotation forest: A new classifier ensemble method. *IEEE Trans. Pattern Anal. Mach. Intell.* 2006, 28, 1619–1630.

[28] Hawkins, D. M., The problem of overfitting. *J. Chem. Inf. Comput. Sci.* 2004, 44, 1–12.

[29] Ying, X., Journal of Physics: Conference Series. IOP Publishing 2019, p. 22022.

[30] Refaeilzadeh, P., Tang, L., Liu, H., Cross-validation. *Encycl. database Syst.* 2009, 5, 532–538.

[31] Chen, T., Guestrin, C., XGBoost: A scalable tree boosting system. *Proc. ACM SIGKDD Int. Conf. Knowl. Discov. Data Min.* 2016, 13-17-Augu, 785–794.

[32] Moore, A. W., Cross-validation for detecting and preventing overfitting. *Sch. Comput. Sci. Carneigie Mellon Univ.* 2001.

[33] Zaremba, W., Sutskever, I., Vinyals, O., Recurrent neural network regularization. *arXiv Prepr. arXiv1409.2329* 2014.

[34] Park, K. Il, Park, M., Fundamentals of Probability and Stochastic Processes with Applications to Communications. Springer 2018.

[35] Madsen, H., Time Series Analysis. 2007.

[36] Palma, W., Long-Memory Time Series. John Wiley & Sons, Inc., Hoboken, NJ, USA 2007.
